# The Effects of Adolescent Idiopathic Scoliosis on Axial Rotation of the Spine: A Study of Twisting Using Surface Topography

**DOI:** 10.3390/children9050670

**Published:** 2022-05-05

**Authors:** Ankush Thakur, Jessica H. Heyer, Emily Wong, Howard J. Hillstrom, Benjamin Groisser, Kira Page, Caroline Gmelich, Matthew E. Cunningham, Roger F. Widmann, M. Timothy Hresko

**Affiliations:** 1 Hospital for Special Surgery Research Institute, Hospital for Special Surgery, New York, NY 10021, USA; thakuran@hss.edu (A.T.); hillstromh@hss.edu (H.J.H.); 2 Department of Pediatric Orthopaedics, Hospital for Special Surgery, New York, NY 10021, USA; heyerj@hss.edu (J.H.H.); emilywg@mit.edu (E.W.); pagek@hss.edu (K.P.); gmelichc@hss.edu (C.G.); widmannr@hss.edu (R.F.W.); 3 Technion—Israel Institute of Technology, Faculty of Mechanical Engineering, Technion Institute, Haifa 320003, Israel; bgroisser@gmail.com; 4 Department of Spine Surgery, Hospital for Special Surgery, New York, NY 10021, USA; cunninghamm@hss.edu; 5 Department of Pediatric Orthopaedics, Boston Children’s Hospital, Boston, MA 02115, USA

**Keywords:** adolescent idiopathic scoliosis, axial rotation, twisting, spine range of motion

## Abstract

Axial twisting of the spine has been previously shown to be affected by scoliosis with decreased motion and asymmetric twisting. Existing methods for evaluating twisting may be cumbersome, unreliable, or require radiation exposure. In this study, we present an automated surface topographic measurement tool to evaluate global axial rotation of the spine, along with two measurements: twisting range of motion (T_ROM_) and twisting asymmetry index (T_ASI_). The aim of this study is to evaluate the impact of scoliosis on axial range of motion. Adolescent idiopathic scoliosis (AIS) patients and asymptomatic controls were scanned in a topographic scanner while twisting maximally to the left and right. T_ROM_ was significantly lower for AIS patients compared to control patients (69.1° vs. 78.5°, *p* = 0.020). T_ASI_ was significantly higher for AIS patients compared to control patients (29.6 vs. 19.8, *p* = 0.023). After stratifying by scoliosis severity, both T_ROM_ and T_ASI_ were significantly different only between control and severe scoliosis patients (Cobb angle > 40°). AIS patients were then divided by their major curve region (thoracic, thoracolumbar, or lumbar). ANOVA and post hoc tests showed that only T_ROM_ is significantly different between thoracic AIS patients and control patients. Thus, we demonstrate that surface topographic scanning can be used to evaluate twisting in AIS patients.

## 1. Introduction

Adolescent idiopathic scoliosis (AIS) is a triplanar deformity that presents with varying sagittal, frontal, and transverse plane components [1]. Scoliosis is defined as a curve of greater than ten degrees in the coronal plane, and is accompanied by vertebral rotation in the axial plane [2]. Axial rotation, or twisting, of the spine is implicated in all motions of daily life, including walking, sitting, standing, and picking up objects off the floor [3,4]. Axial rotation is of even greater importance to athletes who participate in sports such as golf, tennis, lacrosse, and throwing sports [5]. In idiopathic scoliosis, the spine is deformed in the coronal plane and becomes lordotic as it experiences twisting rotation of the vertebral bodies in the axial plane [6,7,8]. The effect that this deformation of the spine has on axial twisting motion has not yet been fully described, and is further complicated by the coupled motion of the spine in the coronal and axial planes [8]. Prior studies have shown that patients with severe lumbar curves have less ability to twist than those without scoliosis [9], and that asymmetric twisting occurs in patients with thoracic scoliosis [10].

Current methods of clinically evaluating axial rotation include those that measure global, lumbar-only, and thoracic-only motion; these techniques include inertia monitors [3], electromagnetic devices [11], low dose CT scans [12], ultrasound [13], a pelvic restraint coupled with a rotameter [14], and goniometers [5]. These methodologies have varying degrees of reliability, and require significant training, cumbersome positioners, or awkward patient positioning [3,6,11,12,13,14]. Surface topography has been previously described as a valid and reliable method for assessing motion in patients with scoliosis, and is a useful tool for evaluating a patient in three dimensions [15,16].

In this study, we present two novel measurements of global axial rotation of the spine using surface topography. Secondary aims of the study include applying these novel measurements to compare subjects with and without idiopathic scoliosis, compare patients with varying severity of scoliosis, and compare patients with curves in different regions of their spine. We hypothesize that there is a difference in twisting motion between people with and without scoliosis, and that this difference is correlated with curve severity. We further hypothesize that thoracic scoliosis has a greater impact on axial motion than lumbar scoliosis.

## 2. Materials and Methods

### 2.1. Subject Recruitment

Subjects were recruited from the Spinal Alignment Registry (SAR). All subjects in the SAR were recruited from the Pediatric Orthopaedic Department at a single institution. The SAR was approved by the institutional review board. Informed consent was obtained for subjects 18 years of age and over, while assent and consent were obtained from subjects and parents for subjects under 18. Patients were defined as subjects aged 11–21 undergoing assessment for adolescent idiopathic scoliosis (AIS) with an EOS biplanar radiograph and with a Cobb angle of at least 10°. Subjects with prior chest wall or spinal surgery, significant medical conditions, and those unable to stand independently or follow instructions were excluded. Controls were recruited from the sports medicine department. Inclusion criteria for controls were subjects 11 to 21 years of age; exclusion criteria were a history of spinal deformity, prior chest wall or spinal surgery, significant medical conditions, or inability to stand independently.

All subjects completed surface topographic scanning, and scoliosis patients received standard of care EOS radiographs.

### 2.2. Surface Topographic Scanning

The 3dMDbody system (3dMD, Atlanta, GA, USA) was used to obtain topographic scans. This system comprises 30 cameras that capture whole body surface topographic scans at 10 frames per second with an exposure time of 1.8 ms per frame, thereby minimizing motion artifacts.

**Figure 1:**
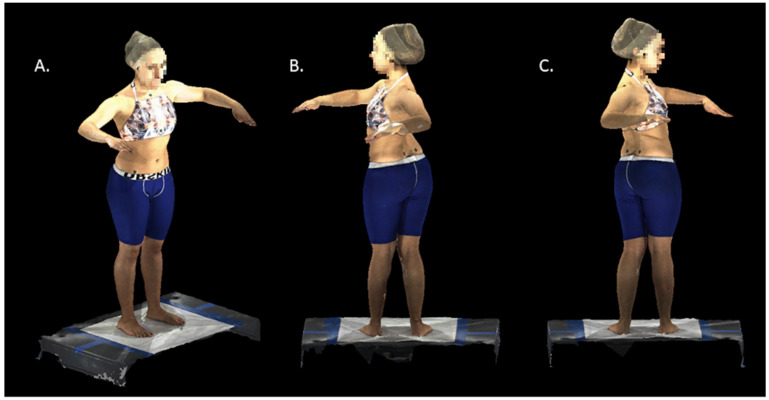
3D Topographic scans: (**A**) forward starting position; (**B**) maximum left twist; (**C**) maximum right twist.

Subjects changed into compression shorts for males and a custom halter top and compression shorts for females. Subjects were placed in the middle of the defined scan area and instructed to march in place and stop to be positioned in their normal angle and comfortable base of stance. Then, they were instructed to elevate their arms and bend their elbows with their forearms forward and palms facing down. The scan was started with each subject facing straight ahead as they were asked to twist maximally to the left and then to the right, holding at each extreme for one second (Figure 1). Subjects were instructed to keep their hips facing forward during the scan. The first frame was selected as the baseline scan, and the maximal left and right frames were used for the twisting measurements.

### 2.3. Scan Processing and Measurements

The raw scans of each subject were processed by a custom analysis pipeline to obtain a torso mesh with full anatomical correspondence between the subjects, as previously described [16]. This allowed us to specify anatomical points once on a torso template; these could then be automatically identified on each scan. Automated measurements were performed with reference to these anatomically significant landmarks, as previously described. The posterior-superior iliac spine (PSIS) and anterior-superior iliac spine (ASIS) were used to normalize each scan to a reference frame defined by the pelvis. The jugular notch and C7 landmarks were used to measure the motion of the torso (i.e., vertebral structures between the sacrum and cervical spine).

**Figure 2:**
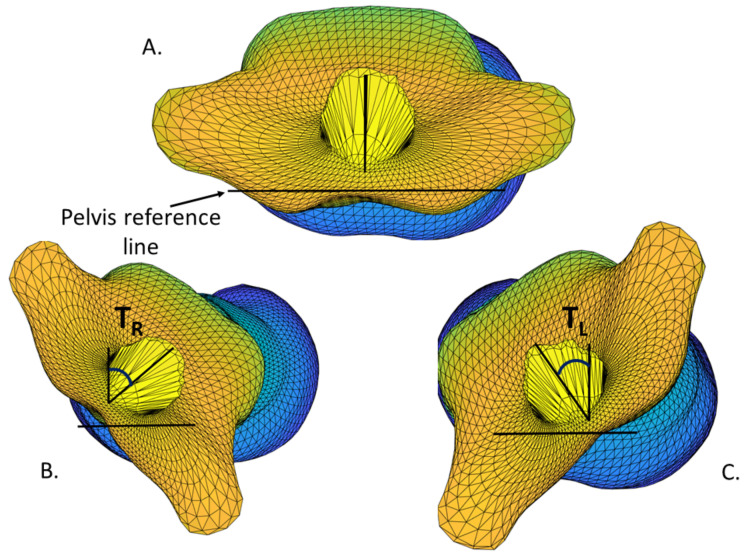
Axial views of torso mesh after alignment to pelvis axis. Twisting angle was measured between the sagittal plane and the line connecting C7 and jugular notch landmarks for each pose: (**A**) forward position; (**B**) maximum right twist; (**C**) maximum left twist.

The twisting angle was defined as the angle between the sagittal plane and the line intersecting the jugular notch and C7 (Figure 2). These twisting angles (maximum left twist and maximum right twist) were measured with respect to the pelvis, as shown in Figure 1 and Figure 2. The twisting range of motion, T_ROM_, was defined as the sum of the maximum left and right twisting angles,

T_ROM_ = T_R_ + T_L_ (1)

where T_R_ is the maximum right twisting angle, and T_L_ is the maximum left twisting angle, with both measured in degrees.

To quantify the differences between left and right twisting, the twisting asymmetry index, T_ASI_, was measured as a percentage, defined as

T_ASI_ = 2 × |T_R_ − T_L_|/(T_R_ + T_L_) × 100 (2)

### 2.4. Statistical Analysis

Independent sample *t*-tests were used to determine whether there were differences in T_ROM_ and T_ASI_ between AIS patients and control patients. A *p* value < 0.05 was considered statistically significant. Subsequently, in order to stratify for curve severity, the AIS subjects were divided into mild scoliosis (10° ≤ Cobb angle ≤ 20°), moderate scoliosis (20° < Cobb angle ≤ 40°), and severe scoliosis (40° < Cobb angle) groups. A one-way ANOVA was used to determine differences across all scoliosis groups and controls for each outcome variable (T_ROM_ and T_ASI_). Pearson correlations were used to evaluate the relationships between Cobb angle and T_ROM_ as well as Cobb angle and T_ASI_. To stratify for curve type, AIS subjects were then divided into groups defined by the region of the apical vertebrae of their largest curve: Thoracic (T2 to T11), Thoracolumbar (T12 and L1), and Lumbar (L2 to L4). A one-way ANOVA was used to determine differences across all scoliosis groups and controls for each outcome variable (T_ROM_ and T_ASI_). For those ANOVAs that were significant, multiple comparison post hoc tests were run to determine which groups were significantly different. Bonferroni correction for multiple comparisons was applied, and a *p* value < 0.0083 was set for significance for post hoc testing based on six unique comparisons. The EOS report provided the maximal axial vertebral rotation (MAVR) for each patient, and Pearson correlation was used to determine the correlation between the maximal axial vertebral rotation (MAVR) and the T_ROM_ and T_ASI_.

## 3. Results

### 3.1. Demographics

This study evaluated 37 controls and 126 patients with AIS. In the control group, there were 20 males (54.1%) with an average BMI of 21.8 kg/m^2^ and average age of 14.2 years. The patient cohort had 51 males (40.5%) with an average BMI of 20.5 kg/m^2^ and average age of 14.6 years (Table 1).

### 3.2. AIS Patients vs. Controls

T_ROM_ was significantly lower for AIS patients compared to controls (69.1° vs. 78.5°, *p* = 0.020) (Table 2). T_ASI_ was significantly higher for AIS patients compared to controls (29.6 vs. 19.8, *p* < 0.023).

### 3.3. Mild, Moderate, and Severe AIS Patients vs. Controls

AIS patients were further divided into mild, moderate, and severe AIS groups. ANOVA demonstrated that T_ROM_ was significantly different (*p* = 0.005) and T_ASI_ was significantly different (*p* = 0.018) between the three groups (Table 3). Post hoc multiple comparison tests demonstrated that differences for both T_ROM_ and T_ASI_ were significant only between controls and severe scoliosis (Table 4). Pearson correlations between Cobb angle and T_ROM_ resulted in a weak relationship, with an R value of 0.233 (*p* = 0.009). Pearson correlations between Cobb angle and T_ASI_ did not show a significant relationship, with an R value of 0.156 (*p* = 0.081).

### 3.4. Thoracic, Thoracolumbar, and Lumbar AIS Patients vs. Controls

AIS patients were divided into thoracic (*n* = 76), thoracolumbar (*n* = 29), and lumbar AIS (*n* = 20) groups. Means for each group are reported in Table 5, and ANOVA demonstrated that T_ROM_ was significantly different (*p* = 0.009) and T_ASI_ was not significantly different (*p* = 0.10) between the three groups. Post hoc multiple comparison tests were run on T_ROM_ only, and demonstrated that differences exist only between controls and Thoracic AIS patients (Table 6).

### 3.5. Comparing T_ROM_ and T_ASI_ to Maximum Axial Vertebral Rotation (MAVR)

The AIS patients’ T_ROM_ and T_ASI_ were compared to each patient’s MAVR as determined by the EOS scan. There was no correlation between MAVR and T_ROM_. There was a statistically significant correlation between MAVR and T_ASI_, although the correlation coefficient was 0.180, indicating a weak correlation. There was a significant and strong correlation between maximum Cobb angle and MAVR (Table 7).

## 4. Discussion

This study presents a new way of measuring axial plane rotation by surface topography, using twisting range of motion and twisting asymmetry index as two values to demonstrate a patient’s motion in the axial plane. We show that scoliosis in the thoracic region limits rotation of the torso in an asymmetric fashion. Our fully automated analysis uses 360-degree Surface Topographic Automated Technology (360 STAT) to normalize patient position to the pelvis, effectively isolating the torso from motion of the hips and legs. Various other methods to evaluate trunk rotation are marred by the necessity for creative solutions to isolate the motion of the spine [3,5,12,14,17]. Many of these methods utilize forward flexion or seated postures to remove the effects of the hips; however, altering the position of the spine into forward flexion affects the twisting ability of the spine, thereby introducing a confounding variable in the measurement of spine rotation [5,11,14,17].

While several methods of measuring axial rotation try to limit their evaluation to either the thoracic or lumbar spine, we believe that global spine motion is more important in the evaluation of overall patient function, as people do not isolate motions to one region of their spine when performing activities of daily life [3,5,14]. Additionally, significant coupling between thoracic motion and lumbar muscle activation has been previously demonstrated, making isolation of motion in one region or the other in vivo nearly impossible [18,19]. While a study by Diers, et al. looked at spine rotation using surface topography, their study looked only at spine rotation involved in ambulation, not maximal efforts [4].

Our study found that there is a significant difference in twisting range of motion as well as twisting asymmetry index between patients with AIS and controls. When AIS patients were stratified by curve severity, we found that a significant difference in both measurements was only found between controls and those with severe scoliosis (> 40° in the coronal plane). The inability to detect differences between controls versus those with mild scoliosis or to detect differences between mild/moderate and moderate/severe patients may be due to the lack of sensitivity of these two measurement tools in detecting smaller differences.

Our work is consistent with various studies that have demonstrated restricted axial plane motion in patients with AIS [9,10]. Sung, et al. evaluated right-handed patients with right thoracic AIS under 40° using surface markers in a motion analysis laboratory to evaluate whether there was a difference in rotation between patients with AIS and controls. They found that patients with AIS had an altered ability to rotate, particularly from left to right (in the direction towards the main thoracic curve). This study by Sung, et al., however, differs from our methodology in its use of fiducial markers and a motion analysis laboratory, as well as in limiting the evaluation to patients with curves under 40° [10]. The 360 STAT system used in the present study does not require markers, and the identification of landmarks is automated. Eyvazov, et al. looked at patients with lumbar AIS curves (Lenke 5) and measured axial rotation using a goniometer; the study found that patients with curves over 40° had less axial plane rotation than those with curves under 40° [9]. Of note, when evaluating all of the different planes of motion, Eyvazov, et al. concluded that only rotation and side bending were associated with curve severity [9]. It has been shown that extension of the spine leads to less axial motion; therefore, the degree of loss of motion may be secondary to the lordotic changes that the spine undergoes in AIS [17].

Uniquely, our study revealed that there was only a difference in rotation between controls and patients with main thoracic curves, not between control patients and those with thoracolumbar or lumbar curves. There are no other studies that have compared rotation of lumbar AIS patients to a control group, as the Eyvazov study did not have a control group for comparison [9]. One explanation of this finding may lie in the differences in the biomechanics of the thoracic and lumbar spines. Studies by Fujimori and Fujii, et al. have demonstrated that axial rotation in the thoracic spine varies between 0.5–2.7° at each vertebral segment, with most motion stemming from the T6-T11 region, while each lumbar spine segment contributed 1.2–1.7° of motion [20,21]. With the largest amount of rotational motion coming from the mid-thoracic spine, it is unsurprising that a derangement in the orientation of the thoracic vertebrae would have a larger impact on the rotational profile than a curve in the lumbar region.

A particularly interesting finding of the present study is that the maximum vertebral rotation as determined by the EOS scan had only a small contribution to the asymmetrical twisting of the spine. This indicates that the change in axial motion in patients with AIS is secondary to other reasons in addition to the anatomical twisting of the spine.

This study has several larger implications which may inspire future research about the importance of axial twisting rotation in the AIS population. Twisting of the vertebral bodies in AIS is believed to have importance in evaluating AIS patients for conservative non-operative care with bracing or scoliosis-specific exercise programs. Equally important, axial motion is considered in preoperative planning, as there are arguments to include a twisting subtype in the Lenke classification [6,7]. Additionally, there is a clear coupling motion between the axial and coronal planes, as it has been shown that the angle of trunk rotation in the axial plane correlates to curve severity in the coronal plane [8]. The next question to be asked is whether we can predict curve severity in the coronal plane by evaluating changes in motion in the axial plane. Further research might compare surface topographic measures of axial rotation prior to treatment with bracing, exercises, or operative care in the AIS patient population; this has already been touched upon by prior studies [22,23,24]. In addition, the 360 STAT system is unique in that it is a surface topographic scanner coupled with an automated system for identifying landmarks on a patient’s body, eliminating the need for placement of fiducial markers.

This study has several limitations. The surface scan system used for this study consisted of multiple high-speed cameras in a controlled environment with custom automated software and measurement algorithms created by the authors, although this method demonstrates the utility of surface topography in the evaluation of motion without the need for ionizing radiation or complex external devices attached to the torso. Additionally, our study may have been underpowered to detect the differences between different severities of scoliosis or in controls versus lumbar or thoracolumbar patients. Furthermore, no mean clinically important difference was found for axial rotation. While we were able to show that there is diminished rotation in patients with scoliosis, we were unable to determine the implications this has on daily life, specific life activities, or sporting abilities, as norms for the degree of axial rotation required for specific activities are not known.

Future studies might look at ways of assessing the impact of rotation in individual sports in order for clinicians to better counsel patients who are diagnosed with AIS as to the impact of AIS on their motion. Another potential future study might include determining the effect of posterior spinal fusion on preoperative versus postoperative twisting motion in patients with AIS. Lastly, in continuing to prospectively evaluate patients, the impact of scoliosis severity and scoliosis region on axial twisting could be reevaluated using a larger number of patients.

In conclusion, we present two new measurement tools for evaluation of axial twisting/rotation. We have demonstrated that there is a significant difference in axial motion in patients with and without scoliosis, and that this difference holds both for those with severe scoliosis compared to controls and with thoracic scoliosis compared to controls.

## Figures and Tables

**Table 1:** Demographics of Control and Patient Groups.

	Controls (*n* = 37)	Patients (*n* = 126)	*p*-Value
Sex			
Male (%)	20 (54.1%)	51 (40.5%)	0.143
BMI, kg/m^2^ (range, SD)	21.8 (16.8–29.7, 3.9)	20.5 (14.2–35.9, 3.8)	0.068
Cobb Angle, ° (range, SD)	n/a	38.3 (9.9–83.1, 19.2)	n/a
Age, years (range, SD)	14.2 (11–20, 2.4)	14.6 (11–21, 2.2)	0.409

BMI: Body mass index, SD: standard deviation; n/a: not applicable.

**Table 2:** T_ROM_ and T_ASI_ of AIS patients and controls and independent sample *t*-test *p*-value.

	Controls (*n* = 37)		AIS Patients (*n* = 126)		
	Mean	SD	Mean	SD	*p*-Value
T_ROM_ (°)	78.5	18.3	69.1	22.0	0.020
T_ASI_ (%)	19.8	17.6	29.6	24.2	0.023

T_ROM_: twisting range of motion, T_ASI_: twisting asymmetry index, SD: standard deviation.

**Table 3:** T_ROM_ and T_ASI_ of mild, moderate, and severe AIS patients and controls with ANOVA *p*-value.

	Controls (*n* = 37)		Mild AIS (*n* = 35)		Moderate AIS (*n* = 30)		Severe AIS (*n* = 61)		*p*-Value
	Mean	SD	Mean	SD	Mean	SD	Mean	SD	
T_ROM_ (°)	78.5	18.3	72.0	22.9	76.1	23.2	64.1	19.9	0.005
T_ASI_ (%)	19.8	17.6	28.4	19.2	22.6	13.8	33.7	29.5	0.018

AIS: adolescent idiopathic scoliosis, T_ROM_: twisting range of motion, T_ASI_: twisting asymmetric index, SD: standard deviation.

**Table 4:** Multiple comparison test of T_ROM_ and T_ASI_ between mild, moderate, and severe AIS patients and controls.

		Mild AIS	Moderate AIS	Severe AIS
T_ROM_	Control	0.190	0.645	0.001 *
	Mild		0.430	0.074
	Moderate			0.010
T_ASI_	Control	0.110	0.620	0.004 *
	Mild		0.303	0.266
	Moderate			0.028

AIS: adolescent idiopathic scoliosis, T_ROM_: twisting range of motion, T_ASI_: twisting asymmetric index. Note: *p*-values < 0.0083 determines significance, based on Bonferroni correction. These values that are significant are denoted by an asterisk (*).

**Table 5:** T_ROM_ and T_ASI_ of thoracic, thoracolumbar, and lumbar AIS patients and controls with ANOVA *p*-value.

	Controls (*n* = 37)		Thoracic AIS Patients (*n* = 77)		Thoracolumbar AIS Patients (*n* = 29)		Lumbar AIS Patients (*n* = 20)		
	Mean	SD	Mean	SD	Mean	SD	Mean	SD	*p*-Value
T_ROM_ (°)	78.5	18.3	65.5	22.9	73.0	21.6	77.4	16.1	0.009
T_ASI_ (%)	19.8	17.6	29.8	27.3	32.1	19.4	25.0	16.9	0.100

AIS: adolescent idiopathic scoliosis, T_ROM_: twisting range of motion, T_ASI_: twisting asymmetric index, SD: standard deviation.

**Table 6:** *p*-Values of the multiple comparison test of T_ROM_ of thoracic, thoracolumbar, and lumbar AIS patients and controls.

		Thoracic	Thoracolumbar	Lumbar
T_ROM_	Control	0.002 *	0.293	0.854
	Thoracic	———————	0.103	0.025
	Thoracolumbar	———————	———————	0.470

AIS: adolescent idiopathic scoliosis, T_ROM_: twisting range of motion. Note: *p*-values < 0.0083 determines significance, based on Bonferroni correction. These values that are significant are denoted by an asterisk (*).

**Table 7:** Correlation of T_ROM_ and T_ASI_ in AIS patients with patients’ maximum axial vertebral rotation.

	MAVR	
	R Value	*p*-Value
T_ROM_	−0.099	0.272
T_ASI_	0.180	0.043
Maximum Cobb Angle	0.762	<0.001

MAVR: Maximum axial vertebral rotation, T_ROM_: twisting range of motion, T_ASI_: twisting asymmetric index.

## Data Availability

The data collected in this study are stored on a secure drive at the Hospital for Special Surgery and can be made available upon request.
